# Alleviation of Rosup-induced oxidative stress in porcine granulosa cells by anthocyanins from red-fleshed apples

**DOI:** 10.1371/journal.pone.0184033

**Published:** 2017-08-29

**Authors:** Ya Xiang, Fangnong Lai, Guifang He, Yapeng Li, Leilei Yang, Wei Shen, Heqiang Huo, Jun Zhu, Hongyi Dai, Yugang Zhang

**Affiliations:** 1 College of Horticulture, Qingdao Agricultural University, Qingdao, China; 2 Qingdao Key Laboratory of Genetic Development and Breeding in Horticultural Plants, Qingdao Agricultural University, Qingdao, China; 3 Key Laboratory of Animal Reproduction and Germplasm Enhancement in Universities of Shandong, Qingdao Agricultural University, Qingdao, China; 4 College of Animal Science and Technology, Qingdao Agricultural University, Qingdao, China; 5 Mid-Florida Research and Education Center, University of Florida, Apopka, FL, United States of America; Zhejiang University, CHINA

## Abstract

Anthocyanins are the polyphenolic phytochemicals which have been shown to scavenge free radicals. In this study, we investigated the effects of anthocyanins extracted from red-fleshed apples *(Malus sieversii)* on reducing oxidative damage by Rosup in porcine granulosa cells (GCs) by measuring intracellular reactive oxygen species (ROS), content of glutathione (GSH), activities of superoxide dismutase (SOD1), catalase (CAT) and glutathione peroxidase (GPX1) and the gene expression of *SOD1*, *CAT*, *GPX1*. Apoptosis was determined with TdT-mediated dUTP-biotin nick end labeling (TUNEL) and apoptosis-related proteins were quantified with Western blotting. The results indicate that Rosup increases oxidative stress by inducing reactive oxygen species production in porcine GCs and the oxidative stress could be reduced by anthocyanins. The gene expression of *SOD1*, *CAT*, *GPX1* and the activities of these enzymes were increased when GCs were treated with anthocyanins and Rosup for 6 hours. Anthocyanins inhibit Rosup-induced apoptosis by increasing expression of antiapoptotic protein Bcl-2 and suppressing the expression of pro-apoptotic protein Bax. Collectively, anthocyanins from red-fleshed apples reduce oxidative stress and inhibit apoptosis in porcine GCs *in vitro*. This approach indicates that antioxidants might be developed from red-fleshed apples.

## Introduction

Apple is one of the most important fruits and a very significant part of human diet. The red coloration of the apple fruit is one of major determinants for its market value as well as its nutritional value [1]. The amount and distribution of anthocyanin determines the color characteristics of the apple peel and flesh [2]. Anthocyanins are a class of water-soluble natural pigments which belong to a subgroup of flavonoids and are ubiquitous in plant flowers and fruits [3]. Anthocyanin is found in plant cell vacuoles of flowers and fruits but also leaves, stems, and roots [4]. Generally, anthocyanin is restricted to the peel of most apple varieties, but it is also enriched in the flesh of red-fleshed apple [5]. The higher level of anthocyanin in red-fleshed apple is thought to provide a rich source of antioxidant for improving health of seed dispersers and to attract them more likely to revisit and disperse more seeds [6]. Anthocyanin has been recognized to have potential health benefits. Increasing evidences suggest that health benefits of anthocyanin are related to their antioxidant activity [7]. As a putative antioxidant, anthocyanin has been reported to effectively scavenge free radicals to protect cells from oxidative stress [8].

The oxidative stress is often caused by redox homeostasis breakdown between ROS particularly free radicals and enzymatic or non-enzymatic antioxidants [9]. The homeostatic imbalance due to overproduction of reactive oxygen species (ROS) and/or a deficiency in antioxidants, particularly under abiotic stress generally induces and exacerbates oxidative stress [10]. Normally, cells produce reactive oxygen species in a balanced manner, however, once the redox balance is disturbed, a chain of peroxidation reactions is triggered to cause functional obstacles in the cytoplasm membrane and to inactivate the normal protein functions [9]. In addition, high level of ROS induces mutations and DNA damage, which may result in mutagenesis and carcinogenesis. For example, ROS leads to the accumulation of 8-oxo-dexoyguanine, a biomarker of cancer in the lung and urine of smokers [11].

Recently the beneficial effects of anthocyanins have been intensively studied [12–16], and its antioxidant properties have been suggested to have potentials for prevention of carcinogenesis [17], reducing mutagens and inhibiting inflammation and allergies [18]. Anthocyanins extracted from purple sweet potato have been reported to maintain the intracellular redox balance of heat-shocked bovine embryos by reducing intracellular ROS and increasing glutathione (GSH) content [19]. You *et al* reported that immature pig oocytes treated with anthocyanins during *in vitro* maturation stimulated *in vitro* development of cloned pig embryos through increasing intracellular GSH and inhibiting ROS [20]. In addition, anthocyanins from fruit of *Nitraria tangutorun* Bobr are able to scavenge free radicals like 1,1-diphenyl-2-picrylhydrazyl (DPPH) and hydroxyl free radical (OH) *in vitro*, and could inhibit lipid peroxidation through improving the activity of superoxide dismutase in rat serum [21].

Apple is a very commonly consumed fruit and is a profound contributor of the phenolic compounds which are very good antioxidants in human body. The apple anthocyanins have been demonstrated to have potential antioxidant activity [22]. However, the anthocyanins used in these studies have been mainly extracted from apple peels, since most apple varieties do not produce anthocyanins in apple flesh compared to apple peels. However, peel is usually removed due to the concerns of pesticide residues on it. In addition, the process for making the apple sauce also requires the removal of apple peel. Therefore, the putative anthocyanins in the apple peel are not consumed by human. However, the red-fleshed apple as indicated by its name contains rich pigments, i.e., anthocyanins, in its flesh. Unlike the anthocyanins from apple peel, little is known about the antioxidant activity of anthocyanins from red-fleshed apple mesocarp.

Ovarian granulosa cells (GCs) play essential roles in the development and maturation of follicles and support oocyte development by producing steroid hormones, estradiol, progesterone and others essential nutrients to the oocytes. Our previous studies suggested that porcine GCs are sensitive to oxidative stress and oxidative stress imposes an adverse effect on GCs. Zhu, et al. demonstrated that exposure to zearalenone greatly results in high level of ROS which inhibits the proliferation of porcine GCs [23], while this inhibition could be alleviated by application of the antioxidant curcumin [9]. Therefore, GCs is a good model for studying cell damage by oxidative stress. Given the potent antioxidant properties of anthocyanins, this study was conducted to investigate whether the anthocyanins extracted from mesocarp of red-fleshed apple could reduce the oxidative stress and the underlying mechanisms.

## Materials and methods

### Anthocyanins extraction

**Fig 1:**
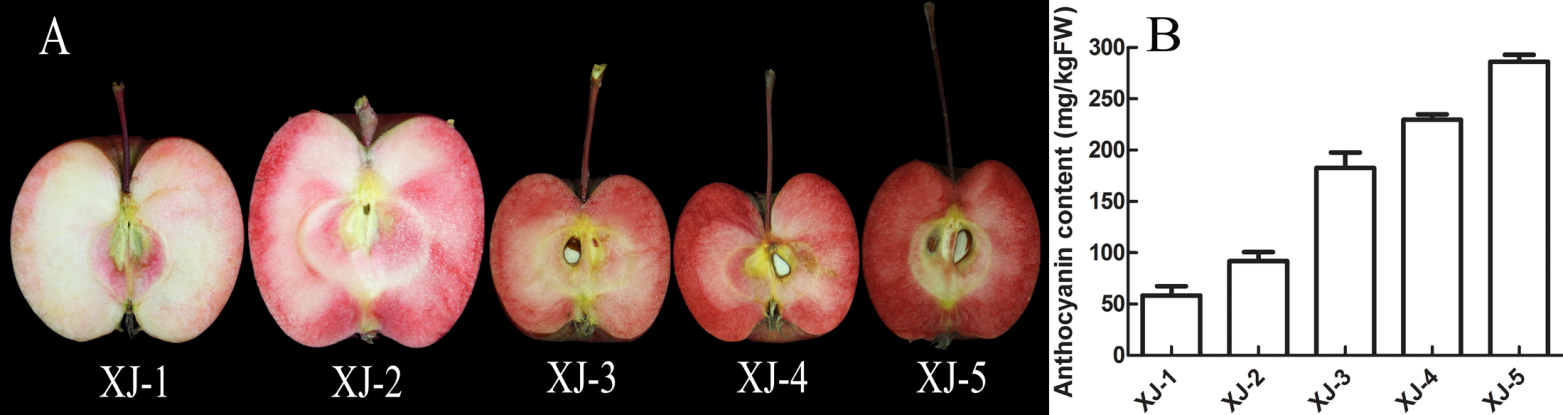
The fruit image and the anthocyanin content in XJ-1~XJ-5 of red-fleshed apple.

Fruits from five different red-fleshed apple varieties (XJ-1, XJ-2, XJ-3, XJ-4 and XJ-5) *(Malus sieversii* f. *neidzwetzkyana* (Dieck) Langenf) with various levels of anthocyanin (Fig 1A) were collected from Experimental Farm of Qingdao Agricultural University (Qingdao, China) and preserved at –80°C in the laboratory. For anthocyanin extraction, samples were ground in liquid nitrogen, and one hundred grams of fruit powder was extracted using 1 L acetone and agitated in an ultrasonic bath (40 kHz/81 W) at 35°C for 30 min. Next, samples were incubated under continuous ultrasonic agitation in the dark at room temperature for 10 h. Then the extract was evaporated to remove acetone at 30°C and filtered through a 0.22 μm micron filter. The purified extract was stored at –20°C for further analysis. Anthocyanins extracted from five different varieties of red-fleshed apples were first tested on their capacity of scavenging free radicals according to the method of Sari et al [24] prior to their further analysis. The anthocyanins with the best capacity of scavenging free radicals were selected for further analysis.

### Determination of total anthocyanin content

Total anthocyanins were determined using a pH differential spectroscopic method [25]. One milliliter of anthocyanin extract was added into 9 ml of potassium chloride buffer (0.025 mmol/L, pH 1.0) or 9 ml of sodium acetate buffer (0.4 mmol/L, pH 4.5). The solutions were measured for their absorbance at 510 and 700 nm. Absorbance of each sample (*A*) was calculated as follows: *A* = (*A*_510_–*A*_700_)_pH 1.0_–(*A*_510_–*A*_700_)_pH 4.5;_ Total anthocyanin content (mg/L) = (*A* × MW × DF × 1 000) / (ε × L) where MW (449.2) is the molecular weight of cyanidin-3-glucoside (predominant anthocyanin in sample); DF is the dilution factor; ε (26,900) was the molar absorptivity; L is the volume of the extraction liquid (n>3).

### Porcine ovaries collection

All animal experimental procedures were conducted in accordance with guidelines and approval of the Ethical Committee of Qingdao Agricultural University. Porcine ovaries were collected from prepubertal gilts at a local slaughterhouse in Qingdao (Shandong, China) and transported to the laboratory within 2 h in 0.9% saline solution containing 100 IU/mL penicillin and 100 μg/mL streptomycin at room temperature.

### Isolation and culture of porcine GCs

Follicular fluid was aspirated from antral follicles (3–6 mm in diameter) using a 20 mL disposable syringe with 18-gauge needle [23]. The fluid was transferred to a conical tube and incubated for 15 min at 37°C before centrifuging at 300 g for 5 min [23], The fluid was transferred to a conical tube and incubated for 15 min at 37°C before centrifugation at 300 g for 5 min [26], and the precipitated pellets (GCs) were aspirated and washed with phosphate buffer saline (PBS) three times. Then GCs were cultured in M199 medium (Hyclone, USA) supplemented with 10% fetal bovine serum (FBS), 100 IU/mL penicillin, 100 μg/mL streptomycin, and 100 IU/mL gentamicin in an incubator at 37°C, 5% CO_2_, at 100% relative humidity [26].

### Treatment of porcine GCs with anthocyanins and Rosup

Porcine GCs at the logarithmic growth phase were plated in 24-well plates with a density of 1×10^5^ cells per well (1 mL) and cultured for 12 h at room temperature. Cells were treated with various concentrations of anthocyanins (0, 20, 60 and 200 μM) for 5.5 h and then Rosup was added into the plate with a final concentration (250 μg/mL) for another 30 min.

### Flow cytometry analysis of ROS

After anthocyanin and/or Rosup treatment, the medium was removed and the cells were incubated with 10 μM 2’,7’-dichlorofluorescein diacetate (DCFH-DA) (in M199 fresh blank medium) in dark at 37°C for 30 min. Fluorescence was measured using a FACSCalibur flow cytometer (Becton Dickinson, New York, USA) at an excitation wavelength of 488 nm and an emission wavelength of 525 nm. Fluorescent signal intensity was recorded and analyzed using CellQuest software (Becton Dickinson, New York, USA). For each sample, 10 000 events were recorded.

### Fluorescent microscopy evaluation of ROS

To further confirm the flow cytometry analysis of ROS, two different probes (Beyotime, Jiangsu, China): DCFH-DA and dihydroethidium (DHE) were used to determine ROS by fluorescent microscopy. Briefly, after anthocyanin and/or Rosup treatments as stated early, the medium were removed and the cells were incubated with 10 μM DCFH-DA or 10 μM DHE (in M199 fresh blank medium) in dark at 37°C for 30 min. Fluorescent intensity was observed and recorded using a fluorescent microscope (Olympus IX-71, Japan), and ROS were measured by mean fluorescent intensity of DCFH-DA or DHE. Image-Pro plus software was used to analyze average fluorescent intensity.

### Gene expression analysis using qRT-PCR

After anthocyanin and/or Rosup treatments, the cells were collected with trypsinization. The mRNA expression of superoxide dismutase (*SOD1*), catalase (*CAT*) and glutathione peroxidase (*GPX1*) in the cells was analyzed by qRT-PCR. Primer3 was used for primers design in this study (Table 1). Total RNA of GCs was extracted using an RNA extraction kit (Aidlab, Beijing, China) according to the manufacturer’s protocols. Total RNA was reverse transcribed to cDNA (Takara, Dalian, China) and gene expression was quantified by real time RT-PCR (LightCycler 480 Real-time PCR System, Germany) using a Light Cycler SYBR Green I Master (Roche, Dalian, China). The reaction system contained 2 μL cDNA, 10 μL SYBR green master mix, 0.4 μL each of primers (10 μM) and 7.2 μL RNase free dH_2_O. Gene expression is presented as 2^−ΔΔCt^. Relative fold changes were calculated and compared to controls (n≥3).

**Table 1:** Primers sets for qRT-PCR.

Genes	Sequences (5'-3')	Accession No.	Fragment size (bp)
*SOD1*	F: ATCAAGAGAGGCACGTTGGA	NM_001190422.1	158
	R: TCTGCCCAAGTCATCTGGTT		
*GPX1*	F: CACCCAGATGAATGAGCTGC	NM_214201.1	163
	R: CATGAAGTTGGGCTCGAACC		
*CAT*	F: AGATGGACACAGGCACATGA	NM_214301.2	172
	R: CCGGATGCCATAGTCAGGAT		
*GAPDH*	F: TCGGAGTGAACGGATTTGGC	NM_001206359.1	147
	R: TGCCGTGGGTGGAATCATAC		

### Enzyme activity measurement

After anthocyanin and/or Rosup treatments as stated early, the cells were collected. The activities of SOD1, CAT, GPX1 and the content of GSH in the cells were measured using kits made form Nanjing Jiancheng Bioengineering Institute (Nanjing, China) according to the manufacturer’s instruction. Absorbance of all reactions was measured using a spectrophotometer at A_450_. Protein concentration was measured using a Thermo Scientific NanoDrop 2000 spectrophotometer at A_280_ (New York, USA) (n≥3).

### TUNEL assay of apoptosis in porcine GCs

Apoptosis was measured by using a TdT-mediated dUTP-biotin nick end labeling (TUNEL) BrightRed apoptosis detection kit (Vazyme Biotech Co., Ltd, Nanjing, China). Briefly, after anthocyanin and/or Rosup treatments as stated early, the medium were removed and GCs were collected and washed in PBS twice, followed by fixation using 4% paraformaldehyde (4% PFA). Then the cells were plated on the pretreated glass slides and dried on a 37°C heating block. Then the slides were washed in PBS three times (5 min each), and incubated with 100 μL proteinase K solution (20 μg/mL) at room temperature for 5 min, and then washed with PBS three times. Then the slides were incubated with 100 μl 1× equilibration buffer sample at room temperature for 30 min, followed by an incubation in a moist chamber with 50 μL terminal deoxynucleotidyl transferase (TdT) incubation buffer (containing 34 μL ddH_2_O, 10 μL 5× equilibration buffer, 5 μL BrightRed labeling mix and 1 μL recombinant TdT enzyme) in dark at 37°C for 60 min. Slides were washed with PBS containing 0.1% Triton X-100 and 5 mg/mL BSA three times and then stained with 10 μg/mL Hochest33342 (Beyotime, Nantong, China) for 5 min, and washed with fresh water three times. Cell samples were examined using a fluorescence microscope equipped with a filter set (620 nm).

### Western blotting

After anthocyanin and/or Rosup treatments as stated early, the medium were removed and the cells were collected and lysed with 20 μl of Radio-Immunoprecipitation Assay (RIPA) for 30 min. The samples were centrifuged at 12,000 rpm for 15 min, and protein concentration was measured using a NanoDrop 2000 spectrophotometer at A_280_ (Thermo scientific, New York, USA). Then the sample was mixed with 5× SDS loading buffer and boiled for 5 min. Forty microgram of protein for each sample was separated using 10% SDS-PAGE and transferred onto the polyvinylidene fluoride membranes. Membranes were washed with 1×TBST (Tris-buffered saline and with 0.1% tween 20) three times for 5 min, then incubated with blocking solution on a shaker at room temperature for 4 h. Then the membrane was washed with 1×TBST five times, followed by incubation overnight at 4°C with Anti-Bcl-2 (1:500), Anti-Bax (1:500) on the shaker. Then the membrane was washed with 1×TBST five times followed by incubation with secondary antibody (1:1,000) for 1 h at room temperature, and developing solution was added prior to visualization.

### Statistical analysis

Each experiment was repeated at least three times. The data are presented as means ± SE. Significant differences among groups were calculated using a Student’s t test or one-way ANOVA. Graph-Pad Prism5 analysis software (San Diego, CA) was used to test multiple comparisons and plot charts.

## Results

### Anthocyanins decreases intracellular ROS in porcine GCs and reduces oxidation induced by Rosup

Five different red-fleshed varieties XJ-1, XJ-2, XJ-3, XJ-4 and XJ-5 with different levels of pigments were used in this study (Fig 1A). The content of anthocyanins in these five varieties were then determined using a pH differential method. The anthocyanin level in these varieties is correlated with their coloration. XJ-5 has strongest red color and highest level of anthocyanins with 286.11 mg/kgFW. By contrast, XJ-1 has a lowest level of anthocyanins (58.45 mg/kgFW), and least red color (Fig 1B). The anthocyanin extract from XJ-5 was then used for the further analysis in this study unless noted.

**Fig 2:**
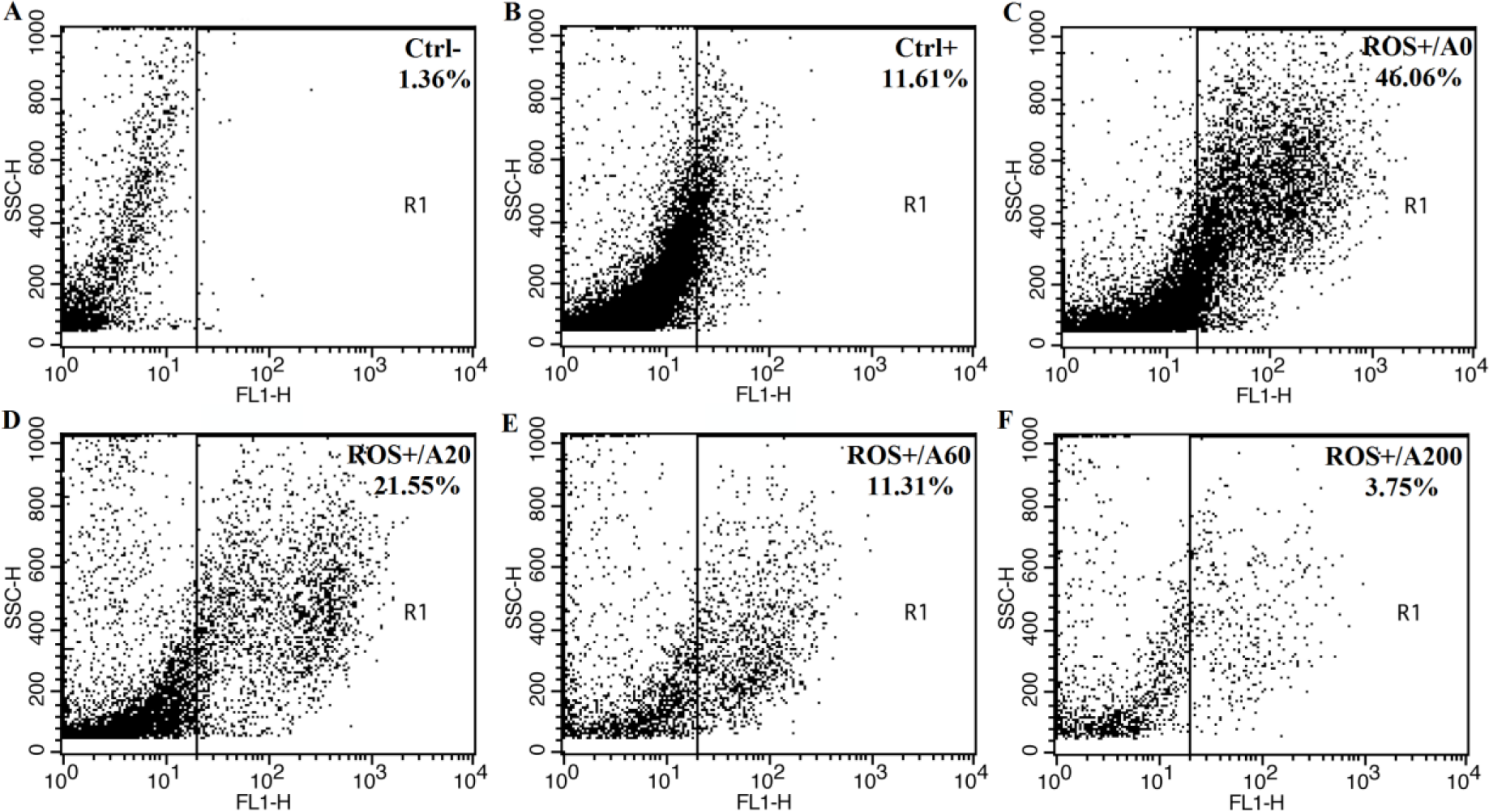
Intracellular reactive oxygen species (ROS) levels assayed with 2’,7’-dichlorofluorescein diacetate (DCFH-DA) fluorescent probe in porcine granulose cells (GCs) treated with Rosup and/or anthocyanins. A. Blank control (untreated with DCFH-DA probe; untreated with Rosup or anthocyanins); B. Negative control (treated with DCFH-DA probe; untreated with Rosup or anthocyanins); C. Positive control and 0 μM anthocyanins (just treated with 250 μg/mL Rosup); D. 20 μM anthocyanins and Rosup (250 μg/mL); E. 60 μM anthocyanins and Rosup (250 μg/mL); F, 200 μM anthocyanins and Rosup (250 μg/mL).

First, we examined the antioxidant activity of anthocyanins from XJ-5 at different concentrations (20, 60 and 200 μM) through testing the capacity of scavenging free radicals by a non-fluorescent dye DCFH-DA by flow cytometry analysis. The level of fluorescent signal is associated with the level of ROS within cells. As shown in Fig 2A, few cells (1.36%) have fluorescent signal which indicates that there is very low signal background in the absence of DCFH-DA probe (Fig 2A). The ROS level in the porcine GCs is relatively low, only 11.61% of cells display signals above the base line (Fig 2B). However, Rosup significantly increases the level of ROS, 46% of cells have a strong fluorescent signal (Fig 2C). Pretreating the cells with 20, 60 and 200 μM of anthocyanins reduce the percentage of cells to 21.55%, 11.31% and 3.75% cells with fluorescent signals (Fig 2D, 2E and 2F) which suggests that anthocyanins from XJ-5 significantly reduce the ROS level induced by Rosup within porcine GCs.

Next, fluorescent microscope was used to confirm the flow cytometry results. Unlike DCFH-DA that is converted to a green fluorescent DCF via oxidation by multiple species of ROS such as hydroxyl radical, carbonate radical and nitrogen dioxide, DHE specifically interacts with superoxide anion to form a red fluorescent product 2-hydroxyethidium which can be detected by a fluorescent microscope with maximum excitation and emission peaks at 518 and 605 nm, respectively.

**Fig 3:**
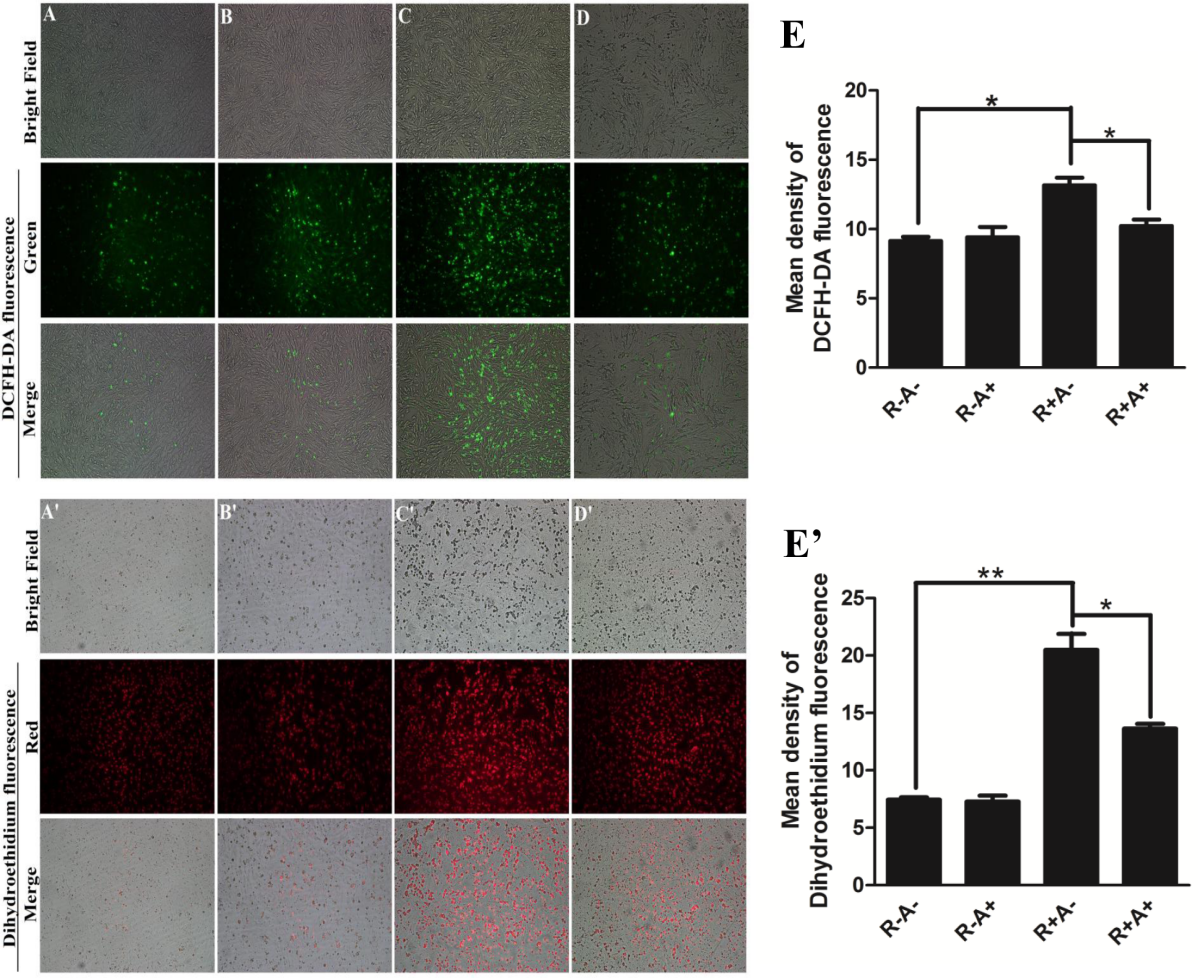
Effect of anthocyanin treatment on intracellular ROS levels assayed with 2’,7’-dichlorofluorescein diacetate (DCFH-DA) and dihydroethidium (DHE) fluorescent probe in porcine granulose cells (GCs). A (A’). Control (untreated with 250 μg/mL Rosup); B (B’). 200 μM anthocyanins; C (C’). 250 μg/mL Rosup; D (D’). 200 μM anthocyanins and 250 μg/mL Rosup; E (E’). Quantitative data for DCFH-DA and DHE. R-A-: no Rosup or anthocyanin treatment (control); R-A+: anthocyanin treatment while no Rosup treatment; R+A-: Rosup treatment while no anthocyanin treatment; R+A+: both Rosup and anthocyanin treatment. The results are expressed as averages ± SE, * indicates P < 0.05, ** indicates P < 0.01.

Interestingly, anthocyanin treatment does not reduce ROS in porcine GCs without Rosup treatment (Fig 3A and 3A’; Fig 3B and 3B’). Rosup induces all types of ROS as revealed by the enhanced fluorescent signals with probe DCFH-DA in Fig 3C and DHE in Fig 3C’. Pretreatment with anthocyanins can significantly alleviate the ROS induction by Rosup in porcine GCs (Fig 3C, 3C’, 3D and 3D’). The quantitative data in Fig 3E (DCF) and Fig 3E’ (DHE) indicate that the fluorescent intensity is reduced by 2.94 and 6.86 a.u. respectively, compared with Rosup treatment only. The data is consistent with the result from flow cytometry analysis (Fig 2F).

### Gene expression of antioxidant enzymes in porcine GCs

**Fig 4:**
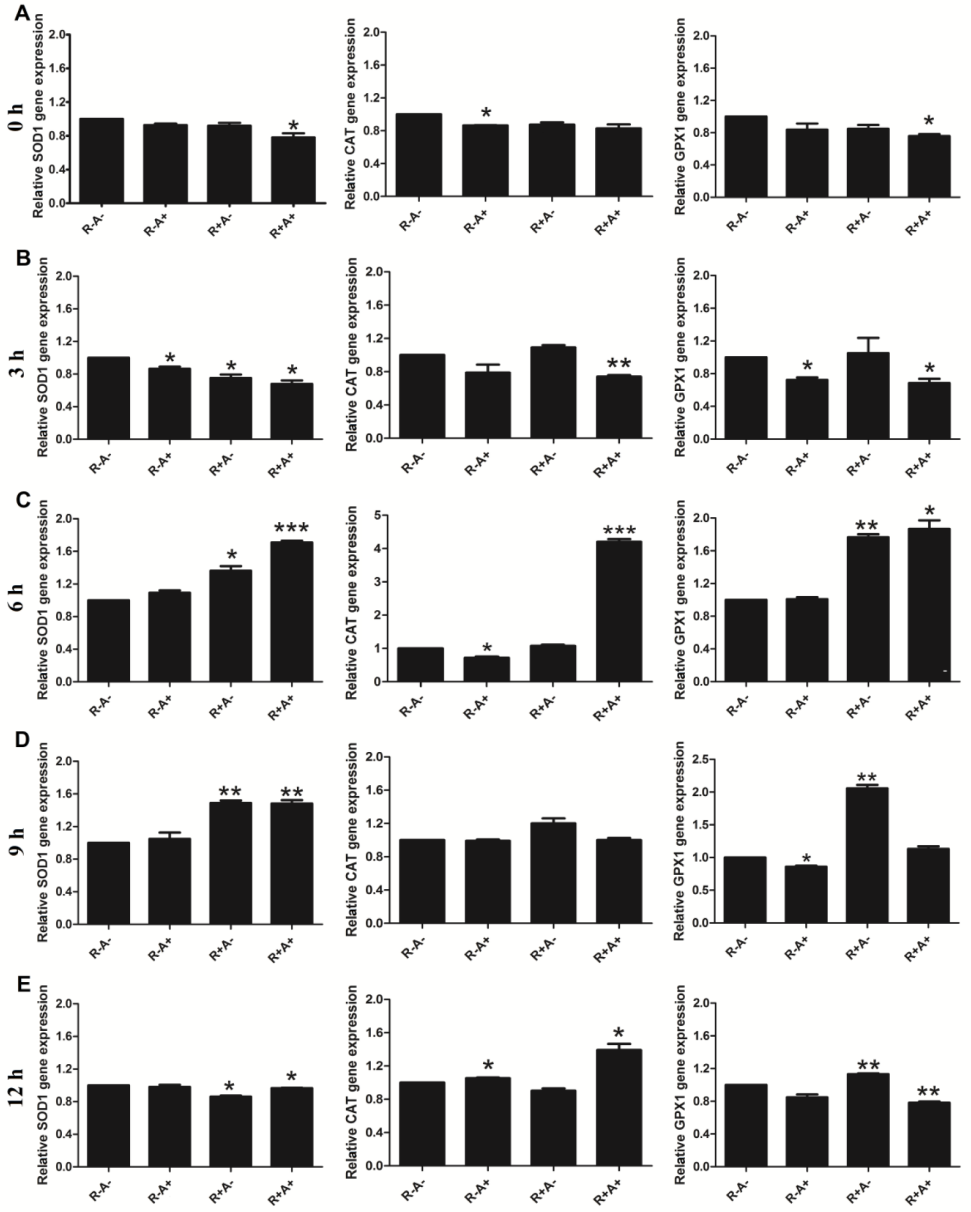
The mRNA levels of superoxide dismutase (*SOD1*), catalase (*CAT*) and glutathione peroxidase (*GPX1*) in porcine granulose cells (GCs) treated with anthocyanins and/or Rosup for different times (0–12 h). A-E. Gene expression in treated cells at 0 h, 3 h, 6 h, 9 h and 12 h. The expression level of β-actin was used as control. R-A-: no Rosup or anthocyanin treatment (control); R-A+: anthocyanin treatment while no Rosup treatment; R+A-: Rosup treatment while no anthocyanin treatment; R+A+: both Rosup and anthocyanin treatment. The relative fold-changes were presented as mean ± SD. Compared to control (R-A-), * indicates P < 0.05, ** indicates P < 0.01.

Given that the potential ROS scavenging capacity by anthocyanins shown above, we next examined the effect of Rosup and/or anthocyanins on gene expression of the antioxidant enzymes in porcine GCs treated with anthocyanins from XJ-5 and/or Rosup for different times. As shown in the Fig 4, gene expression of *SOD1*, *CAT* and *GPX1* are significantly up-regulated in cells treated with anthocyanins and Rosup for six hours (Fig 4B and 4C), although some changes are also observed in cells treated for three hours. Significant increase in expression of *SOD1* and *GPX1* are also observed in the cells treated with Rosup alone for six and nine hours (Fig 4C). However, the gene expression of all tested genes in cells treated with anthocyanin or/and Rosup for twelve hours are not increased as significantly as the ones in cells treated for six and nine hours. These results suggest that the increase in the gene levels of *SOD1*, *CAT* and *GPX1* may be associated with ROS generation.

### Activity of antioxidant enzymes and GSH in porcine GCs

**Fig 5:**
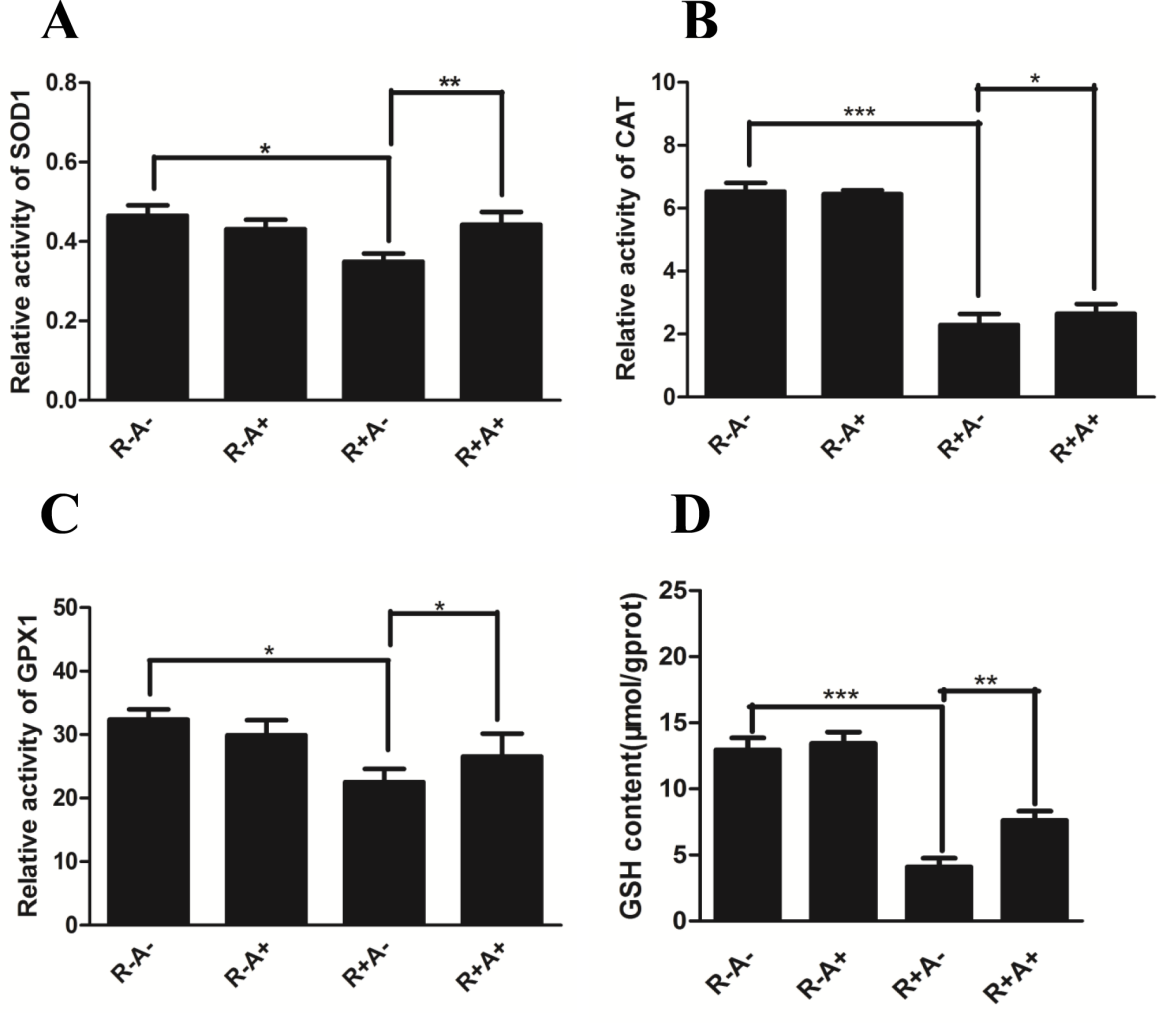
The activity of enzymes superoxide dismutase (SOD1), catalase (CAT) and glutathione peroxidase (GPX1) and the content of glutathione (GSH) in porcine granulose cells (GCs). A. The activity of SOD1; B. The activity of CAT; C. The activity of GPX1; D. GSH content. All values were normalized to protein level and presented as relative fold changes in comparison to untreated control. Data are present as mean ± SE. R-A-: no Rosup or anthocyanin treatment (control); R-A+: anthocyanin treatment while no Rosup treatment; R+A-: Rosup treatment while no anthocyanin treatment; R+A+: both Rosup and anthocyanin treatment. * indicates P < 0.05, ** indicates P < 0.01.

The antioxidant enzymes SOD1, CAT, GPX1 and small peptide GSH are essential for alleviating oxidative stress. To further understand the mechanism underlying the scavenging of ROS by anthocyanins from XJ-5 apples, the activities of these essential antioxidant enzymes were examined. Although anthocyanins have potential antioxidant features, supplementation with anthocyanins alone did not cause great change in the activity of all tested enzymes (Fig 5). Intracellular GSH is decreased by Rosup while it was increased by anthocyanins. Surprisingly, Rosup treatment significantly suppresses the activity of all tested enzymes (Fig 5), however, anthocyanins remarkably rescue the activity of all tested enzymes (Fig 5) which indicates that anthocyanins may exert its action under oxidative stress.

### Effect of anthocyanins on apoptosis of porcine GCs

**Fig 6:**
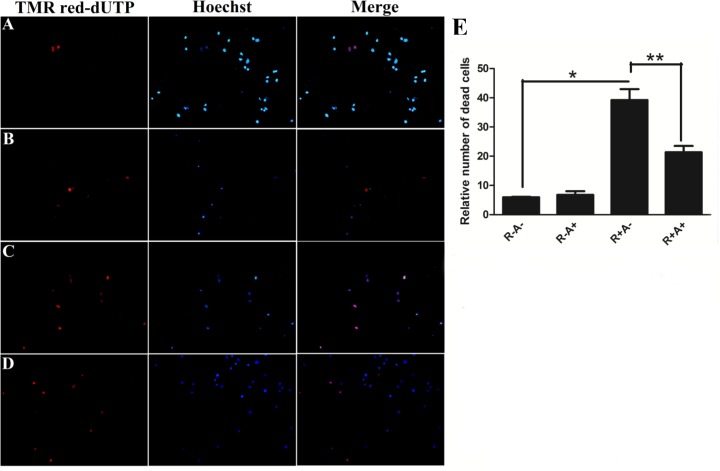
Data for TdT-mediated dUTP-biotin nick end labeling (TUNEL) assay. A. Untreated cells (No Rosup or anthocyanin treatment); B. 200 μM anthocyanin-treated cells; C. 250 μg/mL Rosup-treated cells; D. 200 μM anthocyanin and 250 μg/mL Rosup-treated cells; E. The quantitative data. R-A-: no Rosup or anthocyanin treatment (control); R-A+: anthocyanin treatment while no Rosup treatment; R+A-: Rosup treatment while no anthocyanin treatment; R+A+: both Rosup and anthocyanin treatment. * indicates P < 0.05, ** indicates P < 0.01.

TUNEL staining is widely used for detecting DNA fragmentation by labeling the 3’-OH termini in the dsDNA damage during the programmed cell death or apoptosis. To determine whether ROS induced by Rosup causes apoptosis, TUNEL was applied to detect the DNA fragmentation under Rosup and/or anthocyanin treatments. After treated with Rosup and/or anthocyanins, porcine GCs were stained with TUNEL reagent and counterstained with Hoechst 33342. Unlike TUNEL that only stains apoptotic or dead cells, Hoechst 33342 stains the nuclei of all cells. As shown in Fig 6A, in the absent of oxidative stress the TUNEL positive cells (apoptotic cells) are rarely observed. Rosup induces a remarkable increase of the apoptotic cells (Fig 6C). The TUNEL positive cells (apoptotic cells) are decreased by pretreatment with anthocyanins (Fig 6D). The quantitative data are present in Fig 6E, Rosup dramatically increases the number of apoptotic cells. However, anthocyanins significantly decreased the number of apoptotic cells compared to Rosup. Rosup also alters the morphology of the porcine GCs to cause the cell shrunk, however, anthocyanins can reverse the shrunk morphology caused by Rosup.

**Fig 7:**
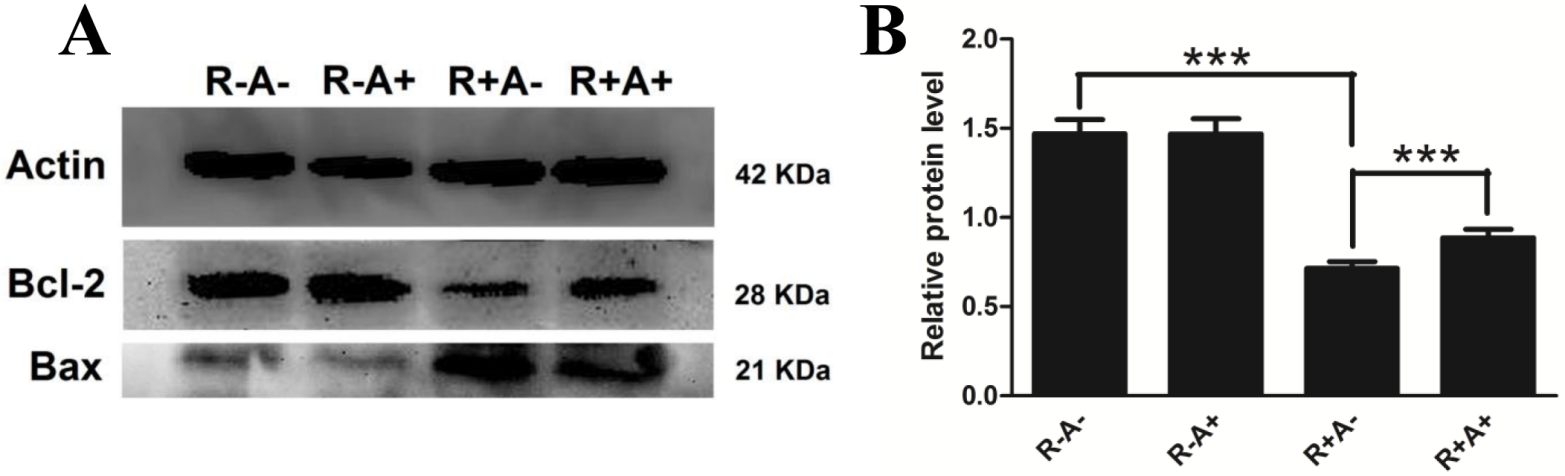
Protein levels of Bcl-2 and Bax in porcine granulose cells (GCs) by Western blotting. A. Images for Bcl-2 and Bax in porcine GCs in different treatments. Actin was used as the loading control. B. The relative protein level (Bcl-2/Bax). R-A-: no Rosup or anthocyanin treatment (control); R-A+: anthocyanin treatment while no Rosup treatment; R+A-: Rosup treatment while no anthocyanin treatment; R+A+: both Rosup and anthocyanin treatment. Compared to the control group, * indicates P < 0.05, ** indicates P < 0.01.

The proteins Bcl-2 (B cell lymphoma/leukemia-2 gene) and Bax are two important members of Bcl-2 family. However, they have different roles in apoptosis. Bcl-2 is considered an important antiapoptotic protein while Bax is believed to be an important pro-apoptotic protein. In order to investigate how anthocyanins decrease the number of apoptotic cells caused by Rosup, the expression of antiapoptotic protein Bcl-2 and proapoptotic protein Bax were determined using Western blotting. Remarkably, Bcl-2 is decreased and Bax is increased by Rosup, while the expression of Bcl-2 is elevated in the pretreatment with anthocyanins (Fig 7A). The relative level of Bcl-2/Bax [27] is a very good indicator of cell survival, in control treatment or anthocyanin treatment the ratio is about 1.50 and it is decreased to 0.76 in Rosup treatment. However, it is increased to 0.94 in anthocyanin and Rosup treatment (Fig 7B) which indicates that anthocyanins could decrease apoptosis induced by Rosup.

## Discussion

ROS contain one or more unpaired electrons in their outermost electronic shell which are very unstable and tended to react with other molecules [28]. ROS at low concentrations acts as second messengers to modulate transcription factors such as NF-κB, p53 and Ap-1 in the signal transduction pathways [29]. On the contrary, overproduction of ROS would lead to oxidative stress [30]. The toxicity of oxidative stress has attracted intensive attention because it could damage cellular proteins and DNA to interrupt their normal functions and to decrease the activity of antioxidant enzymes [31]. Previous studies have reported that oxidative stress can harm human and animal health by causing cell death through DNA breakdown, apoptosis, necrosis and/or protein and lipid degradation [32].

GCs play essential roles in the development and maturation of follicles while these cells are very sensitive to oxidative stress. Our previous study shows that oxidative stress in porcine GCs can be alleviated by curcumin which is the active ingredient of the natural spice curcuma obtained from the root of the plant *Curcuma longa L*. Therefore, porcine GCs were used as a model to explore the role of anthocyanin from red-fleshed apples in alleviation of oxidative stress induced by ROS. However, detailed information regarding ROS induced by Rosup in porcine GCs is limited and it is not clear on the antioxidant effects of anthocyanin extracted from red-fleshed apples.

It has been reported that anthocyanins possess antioxidant properties to reduce ROS *in vivo* [33]. In addition, anthocyanin treatment during *in vitro* maturation (IVM) improves development competence of somatic cell nuclear transfer (SCNT) embryos [20]. However, the ameliorative effects of anthocyanins are largely depended on the concentration [34].

Rosup elevates the oxidative stress by increasing in intracellular ROS level in porcine GCs, and also Rosup results in apoptosis. Surprisingly, anthocyanins, as antioxidants, could scavenge free radicals [24]. Anthocyanins from XJ-5 apple also could reverse Rosup-induced ROS in cultured porcine GCs.

To our knowledge, this is the first study to examine the antioxidant effects of anthocyanin extracted from red-fleshed apple in porcine GCs. Findings of this study are critical to developing possible ways of alleviating the damage caused by oxidative stress *in vitro*.

Free radicals consist of ROS and reactive nitrogen species (RNS). ROS is comprised of not only oxygen-centered radicals such as superoxide anions (O_2_^-^) and hydroxyl radical (·OH), but also non-radical oxygen derivatives such as hydrogen peroxide (H_2_O_2_) and hypochlorous acid (HOCl) [35]. With an overabundance of ROS production and accumulation, oxidative stress occurs and the equilibrated system that keep the balance of oxygen species generation and degradation is compromised which results in oxidative damage to the cells [36, 37]. A favorable environment of the cells should be maintained for providing a complex system to combat with the oxidative stress based on the combination of the various antioxidants and a multitude of enzymes. The expression of genes and the activities of key antioxidant enzymes are increased in the porcine GCs treated with anthocyanins. SOD1 makes O_2_^-^ to H_2_O_2_ and water, CAT and GPX1 also convert H_2_O_2_ to water. Glutathione exits in the forms of a GSH and a disulfide (GSSG) in all living cells [38]. Conversion of GSSG to GSH is catalyzed by GSH reductase, allowing GSH to act as a major intracellular reductant [39, 40]. Studies also suggested that elevated levels of GSH in cells might reduce the occurrence of apoptosis and cell degeneration [41]. The intensity of the reactive oxygen species fluorescence probes DCFH-DA and DHE which have been widely used as indicators to detect the level of intracellular ROS, can freely penetrate cell membrane and be oxidized by intracellular ROS (especially superoxide anion, O_2_^-^) to show green and red signals. The content and variations of ROS can be determined according to how much green and red fluorescence living cells emit. The relative number of apoptotic cells and the pro-apoptotic protein level of Bax are increased in porcine GCs by Rosup, however, the activity of the antioxidant enzymes (SOD1, CAT, GPX1) and the antiapoptotic protein level of Bcl-2 are decreased. While the activity of the antioxidant enzymes and the protein level of Bcl-2 are elevated and the level of ROS, the relative number of apoptotic cells and the protein level of Bax are decreased by anthocyanins.

In current study, Rosup can induce oxidative stress in porcine GCs while anthocyanins from red-fleshed apple could reduce the ROS induced by Rosup and effectively scavenge free radicals such as DPPH, ·OH and O_2_^-^, *etc*., as described in our previous study [42]. When ROS is enriched, oxidative stress occurs and the cellular redox balance is upset. When GCs were pretreated with anthocyanins, the activities of multiple antioxidant enzyme systems were enhanced to defense oxidative stress. In these processes, the anthocyanins execute good protection to the damage by ROS. The severity of the oxidative stress and damage in GCs inhibit nuclear and cytoplasmic maturation and result in apoptosis [43]. Anthocyanins prevent the process by up-regulating the antiapoptosis protein Bcl-2 and down-regulating the pro-apoptosis protein Bax. In summary, our study confirms that anthocyanins could decrease oxidative stress induced by Rosup and inhibit apoptosis in porcine GCs via a pathway of scavenging ROS free radicals and promoting the activities of multiple enzymes systems and gene expression.

## Conclusion

In conclusion, the results of this study suggest that oxidative stress could be induced by Rosup in porcine GCs and the increased oxidative stress could be reduced by anthocyanins extracted from red-fleshed apple, which would expand our understanding of how anthocyanins could alleviate the oxidative stress and provide a theoretical basis for the development of natural antioxidants from red-fleshed apple.
